# The coming of age of insulin-signaling in insects

**DOI:** 10.3389/fphys.2014.00216

**Published:** 2014-06-10

**Authors:** Xanthe Vafopoulou

**Affiliations:** Biology Department, York UniversityToronto, ON, Canada

**Keywords:** insulin-like proteins, interactions of signaling pathways, nutrition and metabolism, growth and development, timekeeping

Since the first isolation of insulin-like proteins (ILPs) from the silkmoth, *Bombyx mori* in 1987, discoveries of ILPs in other insects mushroomed and propelled intense studies of the functional roles of ILPs in numerous insect species. The primary sites of ILP production are brain neuroendocrine cells, but ILPs are also produced in other tissues. Synthesis of ILPs is regulated by many factors including the insulin/insulin-like growth factor signaling pathway (IIS) itself. ILPs can function in an autocrine, paracrine, and endocrine fashion and target a diversity of cells and tissues.

IIS is activated when ILPs bind to the insulin receptor (InR). IIS interacts with various major signaling pathways such as the Target of Rapamycin TOR (an important nutrient sensor) and FOXO (a regulator of stress tolerance, longevity, diapause, and growth). In insects, it also interacts with the signaling pathways of the developmentally significant hormones juvenile hormones (JHs) and ecdysteroids. The numerous interactions of IIS with other major signaling pathways results in a plethora of physiological actions in insects affecting a multitude of life events. The consensus is that the role of IIS in insects bears close similarities to its functional counterparts in the insulin/insulin-like growth factors of vertebrates.

The present Research topic is a compendium of 14 articles on the roles of IIS in a wide spectrum of functions. Of these, seven are reviews focused on different aspects of actions of IIS, five are original research and one is an editorial highlighting the contents of the present Topic. The IIS functions presented here include nutrition, growth and development, behavior, lifespan, semi-lethality, stress, dietary restriction, reproduction, axon guidance, and circadian and seasonal timekeeping. This Research Topic illustrates the complexity of the role of IIS in the detection of environmental cues, their translation into physiological adjustments of insects and the regulation of IIS by other physiological factors involving multiple interactions with other signaling pathways. It also raises stimulating questions for future directions of research in the role of IIS in insect physiology.

A historical account of the discovery of the first ILPs, the bombyxins, in the silkmoth is presented by Mizoguchi and Okamoto (2013). Bombyxins represent a large group of closely related peptides and the chemical characterization of these peptides and their genes as well as their sites of synthesis in the brain is detailed. They function as regulators of sugar homeostasis and promote cell division and tissue growth and interact with ecdysteroids and possibly JH during development. The recent discovery of a single chain bombyxin provided a link between insect ILPs and vertebrate insulin-like growth factors (IGFs).

The role of IIS in *Drosophila* has been extensively studied. Nässel et al. (2013) review the topography of neurons producing ILPs in the *Drosophila* brain (DILPs), their axonal projections and their regulation by factors including neurotransmitters, neuromodulators, hormones, and factors from the fat body. The functional role of DILPs in behavior, lifespan, resistance to stress, and starvation is also discussed. Individual DILPs perform different physiological roles during *Drosophila* development. A review by Kannan and Fridell (2013) pays particular attention to regulation of the differential expression of DILP2, 3, 5, and 6 during development and their regulatory functions in homeostasis, aging, and dietary restriction. DILP2 for example, which is expressed highly in the fly, regulates tissue growth and longevity. High blood levels of ILPs, can also be induced in *Drosophila* by over-expression of DILP2. In an original research article, Sato-Miyata et al. (2014) report that overexpression of DILP2 causes semi-lethality in flies and autophagy of fat body cells that can be rescued by a high protein diet. Li et al. (2014) show that the *Drosophila* insulin receptor (DInR) regulates axon guidance in photoreceptor cells in the developing nervous system by binding to specific adapter protein called Dreadlocks (Dock). However, mutations in DlnR of both putative Dock binding sites did not lead to defects in axon guidance. The authors suggest that Dock may be able to bind to multiple regions of DInR *in vivo* to ensure proper interaction with the receptor in order to achieve appropriate axon guidance.

IIS is also involved in regulation of female reproduction in conjunction with the JH and ecdysteroid signaling pathways. Badisco et al. (2013) review this extensively investigated area and summarize the roles of IIS in regulation of vitellogenesis, oogenesis, reproductive diapause, caste differentiation, and division of reproductive labor in various insects, primarily *Drosophila*. An insect also used as a model animal to study the integration of nutritional information and reproduction, is the mosquito. Hansen et al. (2014) review the complex interactions and cross-talk between complex signaling networks involving JH, ecdysteroids, nutrients, and IIS/TOR in the regulation of yolk protein precursor gene expression in mosquitoes.

IIS is also involved in the regulation of circadian and seasonal timekeeping at multiple levels of physiological organization. The relationship between metabolic state and the circadian clock in the brain is reviewed by Erion and Sehgal (2013). In *Drosophila*, nutrition and IIS influence the molecular oscillator in brain clock cells, thereby modulating daily activity rhythms and consequently affecting many key survival behaviors like feeding, sleep, reproduction, and maintenance of circadian rhythms. Vafopoulou and Steel (2014) report that *in vitro* treatment with bombyxin or prothoracicotropic hormone (PTTH) induces expression of the clock protein PERIOD in *Rhodnius* circadian clock cells, demonstrating a direct effect of these hormones on the molecular oscillator and indicating interaction of the insulin and PTTH signaling pathways in the regulation of clock genes. The article also shows that the clock cells that control rhythmicity in hormones are themselves responsive to feedback from these same hormones by the IIS and PTTH signaling pathways. Sim and Denlinger (2013) review that the IIS/FOXO pathways influence many aspects of diapause, such as arrest of cell cycle and development, interference with life span, suppression of metabolism, fat body hypertrophy, enhanced stress tolerance, and probably enhanced innate immunity in various insects and in *C. elegans*. IIS involvement in diapause is executed in association with the JH signaling pathway.

Smith et al. (2014) investigate the possible involvement of IIS in the regulation of steroid synthesis by the prothoracic glands of *Manduca*. Unlike *Bombyx*, in which this process involves intersection of the IIS pathway with the PTTH signaling pathway, both bombyxin and *Manduca* ILP fail to stimulate steroid synthesis in *Manduca*. The authors conclude that steroid synthesis in *Manduca* is dependent on PTTH signaling and independent of IIS and does not require interaction of these two pathways.

The extensive variability of tissue responses to IIS raises the question of how and why insect tissues respond so differently to insulin. This is addressed in the review by Koyama et al. (2013). Based mainly on studies in *Drosophila*, but also in other insects, the authors summarize evidence that the IIS/TOR pathways regulate plasticity of organ growth and size during development. This plasticity depends on interactions between IIS/TOR pathway with those of ecdysteroid and JH, but that there are also differences in tissue sensitivities to the pathways.

In conclusion, IIS forms complex networks with several other signaling pathways. This complexity of pathway interplay and crossover introduces numerous variables that are difficult to determine or control during an experiment. Nijhout and Callier (2013) developed a scaled mathematical model of the insulin/TOR/MAPK signaling network that controls tissue growth that is a valuable tool to use when investigating the qualitative properties of this network.

## Conflict of interest statement

The author declares that the research was conducted in the absence of any commercial or financial relationships that could be construed as a potential conflict of interest.
